# The Complete Genomic Sequence of Pepper *Yellow Leaf Curl Virus* (PYLCV) and Its Implications for Our Understanding of Evolution Dynamics in the Genus *Polerovirus*

**DOI:** 10.1371/journal.pone.0070722

**Published:** 2013-07-30

**Authors:** Aviv Dombrovsky, Eyal Glanz, Oded Lachman, Noa Sela, Adi Doron-Faigenboim, Yehezkel Antignus

**Affiliations:** Department of Plant Pathology, ARO, the Volcani Center, Bet Dagan, Israel; Volcani Center, Israel

## Abstract

We determined the complete sequence and organization of the genome of a putative member of the genus *Polerovirus* tentatively named *Pepper yellow leaf curl virus* (PYLCV). PYLCV has a wider host range than *Tobacco vein-distorting virus* (TVDV) and has a close serological relationship with *Cucurbit aphid-borne yellows virus* (CABYV) (both poleroviruses). The extracted viral RNA was subjected to SOLiD next-generation sequence analysis and used as a template for reverse transcription synthesis, which was followed by PCR amplification. The ssRNA genome of PYLCV includes 6,028 nucleotides encoding six open reading frames (ORFs), which is typical of the genus *Polerovirus*. Comparisons of the deduced amino acid sequences of the PYLCV ORFs 2-4 and ORF5, indicate that there are high levels of similarity between these sequences to ORFs 2-4 of TVDV (84-93%) and to ORF5 of CABYV (87%). Both PYLCV and *Pepper vein yellowing virus* (PeVYV) contain sequences that point to a common ancestral polerovirus. The recombination breakpoint which is located at CABYV ORF3, which encodes the viral coat protein (CP), may explain the CABYV-like sequences found in the genomes of the pepper infecting viruses PYLCV and PeVYV. Two additional regions unique to PYLCV (PY1 and PY2) were identified between nucleotides 4,962 and 5,061 (ORF 5) and between positions 5,866 and 6,028 in the 3' NCR. Sequence analysis of the pepper-infecting PeVYV revealed three unique regions (Pe1-Pe3) with no similarity to other members of the genus *Polerovirus*. Genomic analyses of PYLCV and PeVYV suggest that the speciation of these viruses occurred through putative recombination event(s) between poleroviruses co-infecting a common host(s), resulting in the emergence of PYLCV, a novel pathogen with a wider host range.

## Introduction

Pepper ( *Capsicum* *annum* ) is an economically important crop worldwide including Israel, where about 3,000 hectares are grown year round for the local and export markets. Since 1998, a viral disease has been found in pepper crops in the south and east of Israel, causing serious economic damage. The disease has been observed mostly in open fields and greenhouses covered with low mesh nets through which insects can easily pass. Disease incidence is higher in greenhouses in which an integrated pest management (IPM) regime or biological control methods are used. The disease symptoms include shortening of stem internodes, inter-veinal yellowing, upward curling of the leaf blade and small, discolored fruit. The disease-causing agent was tentatively named *Pepper yellow leaf curl virus* (PYLCV) [1,2,3]. PYLCV is transmitted by grafting and is also transmitted in a persistent manner by two aphid vectors, *Aphis gossypii* and *Myzus* *persicae* [1,2,3]. Reports from Turkey and Tunisia [4] and more recently from India, Indonesia, Mali, Philippines, Taiwan, and Thailand [5] inform of pepper crops showing symptoms similar to those found in PYLCV-infected pepper crops. According to the 2011 report of the International Committee on Taxonomy of Viruses, http://www.ictvonline.org/virusTaxonomy.asp?version=2011&bhcp=1, the family *Luteoviridae* is composed of three genera: *Enamovirus* (one species), *Luteovirus* (6 species), *Polerovirus* (13 species) and unassigned viruses (8 species). Luteoviruses and Poleroviruses are exclusively transmitted by aphids in a persistent (circulative) and nonpropagative manner [6,7]. Serological analysis, the morphology of the virions, disease symptoms and the partial sequences of the coat protein and movement protein indicate that PYLCV is a putative member of the genus *Polerovirus* (family: *Luteoviridae*) [1,2,3].

Yellowing symptoms on pepper plants caused by *Capsicum yellows virus* (CYV) [8] and *Pepper vein yellows virus* (PeVYV) [9] have been reported in Australia and Japan, respectively. Both of these viruses were assigned to the genus *Luteovirus*, based on particle morphology, serology and aphid transmission, but not on any sequence information. Recently, the complete genomic sequence of PeVYV was determined and examination of this sequence led to PeVYV being classified as a new polerovirus [10]. Another pepper-infecting polerovirus, *Pepper yellows virus* (PepYV), was identified recently in Turkey and has been partially sequenced (accession no. FN600344). The goal of the present study was to obtain the complete genomic sequence of PYLCV and to establish its systematic and evolutionary position among the pepper-infecting members of the genus *Polerovirus*.

## Materials and Methods

### Maintenance of virus cultures and virus purification

PYLCV-infected pepper plants served as the virus source in this study. Virus cultures were renewed monthly, using the green peach aphid *Myzus* *persicae* (Sulzer) as the vector for the inoculation of 3 to 4 week old pepper plants (*C. annuum* cv. Maor). To study virus transmission, we allowed 24 h of acquisition access feeding (AAF), followed by 48 h of inoculation access feeding (IAF). At the end of the IAF period, plants were sprayed with the insecticide imidacloprid (Confidor, Bayer, Leverkusen, Germany) before being transferred to a growth chamber to allow symptoms to develop. Virions were purified from fresh leaf tissue harvested from infected pepper plants. Purification was carried out essentially as described by Rowhani and Stace-Smith (1979) [11]. Samples containing the purified viral particles were stained with 1% uranyl acetate before being analyzed by transmission electron microscopy (TEM) (Tecnai G2, FEI-Philips, Netherlands).

### Extraction and characterization of viral RNA

Purified virion preparations served as source material for RNA extractions, as described previously [12]. Virion preparations were incubated with RQ RNase-free DNase I (Promega, Madison, WI, USA) for 1 h at 37°C, and then with Proteinase K (PK) (Sigma, St. Louis, MO, USA) at a final concentration of 200 µg/ml for 1 h at 37°C. The viral nucleic acid was further purified and precipitated with acidic phenol (Ambion/Applied Biosystems, Foster, CA, USA). The aqueous phase of the preparation was precipitated overnight at -20°C in the presence of glycogen (Fermentas, Burlington, Canada), 0.1 M sodium acetate and 3-4 volumes of Isopropanol. The precipitated viral RNA was washed twice with 75% ethanol and allowed to air dry for 10 min. The dry viral RNA was stored at -80°C for further analysis. Further purification of the viral RNA was performed by agarose gel electrophoresis, in which the RNA solution dissolved in 1% Tris-borate EDTA was loaded onto a 1% (TBE) agarose gel and the electrophoresis was carried out at 90 V for 1 h.

### Reverse transcription (RT) and PCR amplification

Virion RNA served as a template for the RT reaction carried out using the Verso cDNA kit (Thermo, Fisher Scientific, San Jose, CA, USA) or the Maxima Reverse Transcriptase kit (Fermentas). Sequence-specific complementary primers were used in the reaction. The resulting cDNA was amplified in a PCR reaction using Taq polymerase (DreamTaq; Fermentas) or Advantage 2 Polymerase Mix (Clontech-Takara Bio, Madison, WI, USA) and specific primers flanking the PYLCV genes, which are shown in Table 1.

**Table 1:** List of selected primers used for the amplification and identification of the entire *Pepper yellow leaf curl virus* (PYLCV) genome.

**No.**	**Name (position)**	**Orientation**	**Sequence (5’-----3’)**
**1**	PY-F1 (1-30)	sense	ACAAAATATACGAAGAGAGAGAGCCCTTGC
**2**	PY-F16 (16-35)	sense	AGAGAGAGCCCTTGCTAGTG
**3**	PY-TV-F-198 (198-217)	sense	TGCTCTATTTGTGCTCTCCT
**4**	PY-F593 (593-616)	sense	TCTGGGTCATTGTCTCCTGGACCT
**5**	PY-F944 (944-966)	sense	TGACCTGTCACCACGTGGGCACA
**7**	PY-R1033 (1014-1033)	antisense	GTGTGAATAGGCGGAGTGGT
**9**	PY-F1455 (1455-1474)	sense	CGTCACCTCATATCCCCAGT
**10**	PY-F2679 (2679-2698)	sense	CGAGAAATCGCTCTTTGGAG
**11**	PY-TV-F2972 (2972-2991)	sense	TGTCCTGTGTGTGAGCGATG
**12**	PY-F3802 (3802-3822)	sense	GGAGGAAGGTC**R**AGCAACAGC
**13**	PY-F-3826-3846	sense	ACTTTCATCTTCAACAAGGAC
**14**	PY-TV-R-3531-3550	antisense	AAATCCTGACGCAAATCCTG
**15**	PY-F-3973-3992	sense	GTCAGCGAATCCTCTTCCAC
**16**	PY-CA-F-4715-4734	sense	CGAAAATGAATGGCAAATCC
**17**	PY-Pe-R-5633-5652	antisense	CTGCTGTCTTTGAGCTGTAG
**18**	PY-CA-R-4765-4784	antisense	AGTTCGAAAGAAGCGAACCA
**19**	PY-d-R-4219-4242	antisense	CTATTTGGGGTTGTG**Y**A**R**TTGCAC
**20**	PY-d-R-4186-4205	antisense	TGGAAAAA**Y**CC**R**GCGGCAAC
**21**	PY-Pe-R-5883-5902	antisense	ACTGGACCGCTGATTTTACG
**22**	PY-Pe-R-6001-6028	antisense	AATTCACTAGTGATTGGGGGGGTATCTA

### Synthesis of double-stranded (ds) cDNA, cloning and sequence analysis

cDNA was synthesized in Verso enzyme mix in the presence of PYLCV-specific primers designed to obtain contigs. Second strand synthesis was performed using the Universal RiboClone cDNA Synthesis System (Promega) according to the manufacturer’s instructions. The ds cDNA was purified using a PCR purification kit (Zymo research, Irvine, CA, USA) and the obtained double-stranded fragments were cloned into pUC19 after digestion by *Sma*I and dephosphorylation (Fermentas). The resulting recombinant plasmids were sequenced to identify viral genomic sequences.

PCR products were cloned into the pGEM-T-easy vector (Promega). Plasmid DNA was extracted with a plasmid extraction kit (Promega). Both strands of each of the cloned cDNA fragments were sequenced by HyLab Sequencing Service (Rehovot, Israel). Nucleotide sequence analysis was carried out using DNAMAN (Lynnon BioSoft) and software from the NCBI database. The obtained complete genomic sequence of PYLCV was appended to the existing sequence in GenBank (accession number HM439608).

### Next-generation sequencing (NGS) via the SOLiD system

The PYLCV RNA band was excised from the gel and eluted with TBE buffer using a mini-GEBA flex-tube dialysis kit. Then, the viral RNA was sequenced using the SOLiD version 3 instrument [13], following Applied Biosystems’ protocols, at the center for Genomic Technologies at the Hebrew University of Jerusalem, Israel. Libraries were prepared using SOLiD Total RNA-Seq Kit (Applied Biosystems), following the whole transcriptome protocol. Size selection was performed using E-Gel EX Agarose Gels (Invitrogen\ Life Technologies, Grand Island, NY, USA). Sample fragmentation, library fragment size and sample purification were analyzed using the Agilent 2100 Bioanalyzer (Agilent Technologies, Santa Clara, CA, USA). The library was quantified using the KAPA ABI SOLiD Library Quantification Kit (KAPA Biosystems, Woburn, MA, USA).

### Genome assembly

**Figure 1:**
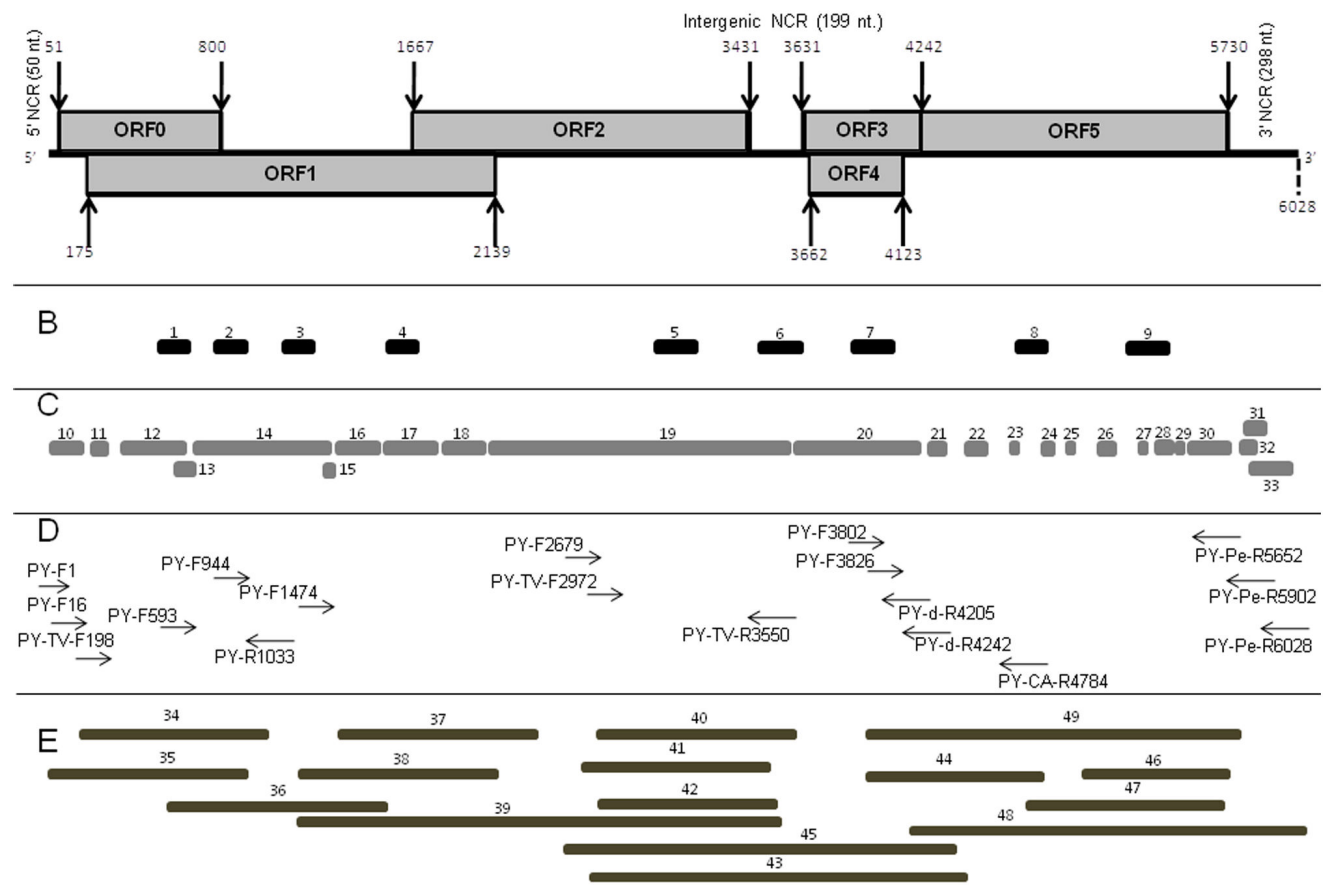
Schematic presentation of the genome organization of *>Pepper yellow leaf curl virus* (PYLCV). **A**. The grey boxes represent the main predicted ORFs. The positions of the start and termination codons of each ORF are marked with arrows and the numbers at the base of each arrow indicate the nucleotide position at the borders of each ORF. **B**. The bold lines (1-9) show the location and size of the NGS SOLiD contigs obtained via *de novo* analysis. **C**. The NGS SOLiD contigs (10-33) obtained via whole genome reference assembly analysis using *Tobacco vein-distorting virus* (TVDV), *Cucurbit aphid-borne yellows virus* (CABYV) and *Pepper vein yellowing virus* (PeVYV) genomes as references (GenBank accession numbers EF529624, AY529654 and AB594828, respectively). **D**. Selected primers that were used for reverse transcription (RT); ds cDNA synthesis and PCR amplification (see Table 1). **E**. Selected clones (34-43) that were used to map the complete PYLCV genome (GenBank accession number HM439608).

The NGS bioinformatic analysis was carried out at the Goldyne Savad Institute of Gene Therapy at the Hadassah Medical Center at Jerusalem, Israel. NGS sequence analysis and assemblies were performed using two sequential bioinformatic approaches. First the *de novo* contig was assembled using the SOLiD System *de novo* Accessory Tools 2.0 pipeline (http://solidsoftwaretools.com/gf/project/denovo/). The short-read assembler at the core of this pipeline is the color space aware version of Velvet [14]. Second, we used the whole genome reference assembly analysis that was performed by comparisons with selected sequences of TVDV, *Cucurbit aphid-borne yellows virus* (CABYV) and PeVYV (GenBank accession numbers EF529624, AY529654 and AB594828, respectively). The SOLiD System Analysis Pipeline Tool (Corona Lite, http://solidsoftwaretools.com/gf/project/corona/) was used to align the obtained reads with the initial, partial PYLCV reference sequence and the complete genomic sequences of TVDV, CABYV and PeVYV. Given the high level of polymorphism within viral species, a maximum of 6 mismatches per read were allowed in each alignment. The combination of the above mentioned bioinformatic approaches allowed the identification of most of the PYLCV genome, excluding a few sequence gaps. Verification of the sequence gaps and the authenticity of the NGS data was accomplished by two procedures: RT-PCR amplification using sequence-specific primers and RNA extracted from purified PYLCV virions that served as a template and by synthesis, cloning and sequencing of PYLCV ds cDNA fragments (Figure 1, A-E).

### Bioinformatic sequence analysis and phylogeny

Sequence homology was determined using the Basic Local Alignment Search Tool (BLAST; http://blast.ncbi.nlm.nih.gov/Blast.cgi). Multiple sequence alignments were analyzed using the BioEdit and ClustalX software programs. Phylogenetic tree predictions were carried out using ORF1-ORF5 amino acid sequences selected from five diferent poleroviruses. First, the MUSCLE program was used to align each ORF [15]. Then, phylogenetic trees were constructed for each data set based on a ML framework, using the PhyML software with 100 bootstrap replicates [16]. To detect recombination event within the genomes of PeVYV and PYLCV, we compared each of these genomes to those of TVDV, CABYV and *Potato leaf roll virus* (PLRV) using the RDP3 package (http://darwin.uvigo.es/rdp/rdp.html) [17]. Detection of recombination was performed by the following methods: RDP [18], GENECONV [19], Bootscan [20], MaxChi [21], Chimera [22] and 3seq [23] employing the default parameters. To increase reliability, only recombination signals detected successfully by all of the abovementioned methods were considered real (*p*-value < 0.05). Additional analysis of recombination event was done using RAT (Recombination Analysis Tool) [24].

## Results

### Isolation of viral RNA and Next-generation sequencing (NGS) (SOLiD) analysis

In a previous study, separation of the viral RNA extracted from purified virions on TBE agarose gels revealed the presence of an RNA fraction with an estimated size of ~6.5 kb [3]. Currently, the PYLCV RNA band was excised from the gel and was sequenced using the NGS SOLiD technology. The obtained sequence was validated later by RT-PCR amplification and by ds-cDNA synthesis, followed by cloning and the classical Sanger sequencing.

The analysis of the sequencing information obtained using several different methods are described in Figure 1. The complete genome of PYLCV is composed of 6,028 nucleotides (nts) and has a typical polerovirus genome organization that includes six open reading frames (ORFs) (Figure 1). ORF0 encodes protein 0 (P0), which has a predicted molecular weight (MW) of 28.14 kDa. ORF1 encodes protein 1 (P1), which has a predicted MW of 72.12 kDa. ORF2 encodes the 65.58 kDa protein 2 (P2), the putative RNA-dependent RNA polymerase (RdRp). ORF3 encodes the 22.51 kDa coat protein (CP) and overlaps ORF4, which encodes the movement protein. ORF5 has a read-through domain (RTD) that yields a fusion protein with a predicted MW of 77.94 kDa, which is composed of the products of ORF3 (CP) and the adjacent ORF5 (Figure 1).

**Figure 2:**
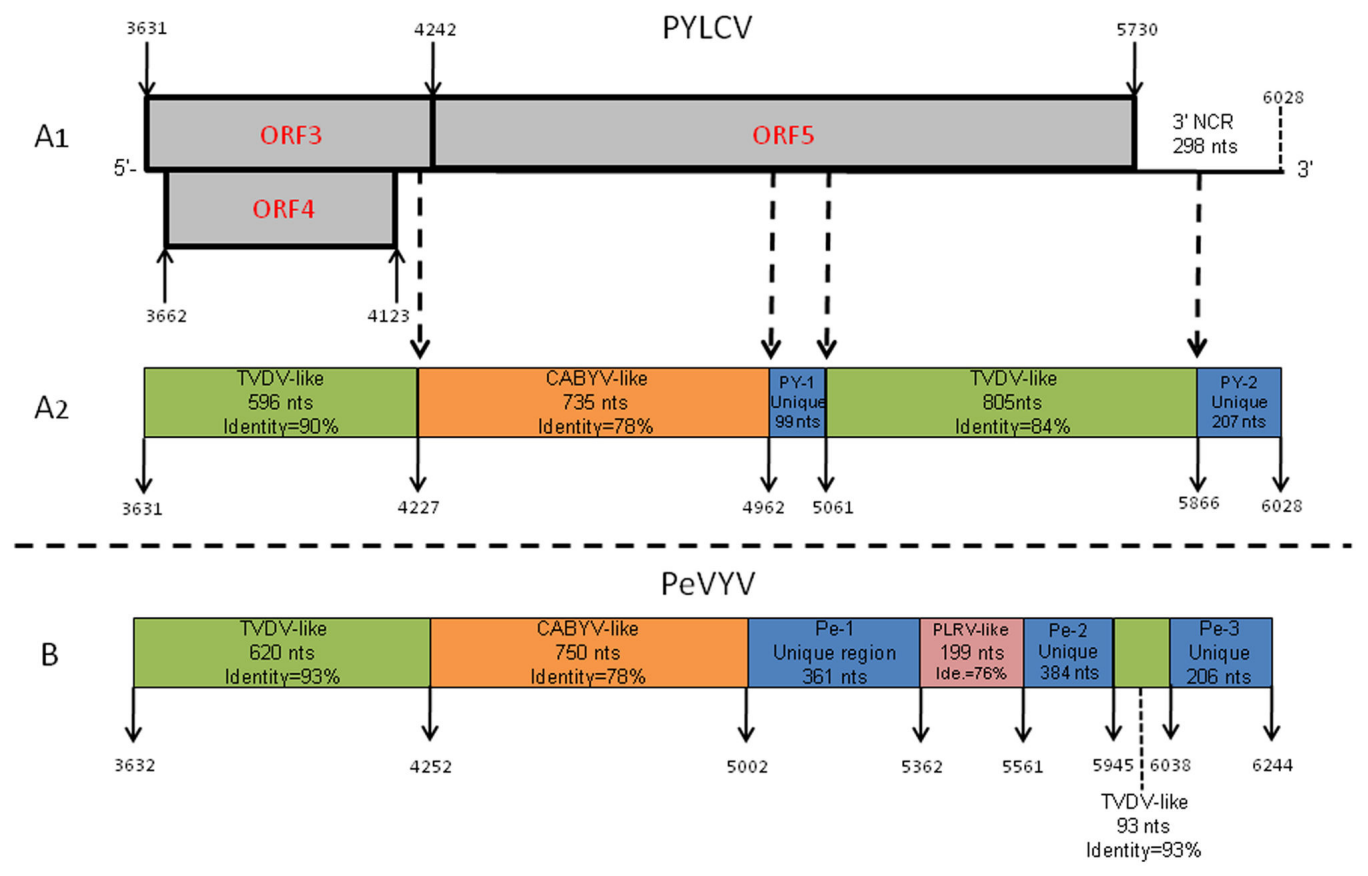
Schematic presentation of the putative recombination regions in the 3' end half of the genomes of the pepper-infecting poleroviruses *Pepper yellow leaf curl virus* (PYLCV) and *Pepper vein yellowing virus* (PeVYV). (**A1**) The 3' part of the PYLCV genome, including its adjacent ORFs. (**A2**) A scheme describing the distribution of nucleotide sequences in PYLCV genome, which illustrates the high level of similarity of the PYLCV sequences with sequences found in the homologous ORFs of the two poleroviruses *Tobacco vein-distorting virus* (TVDV) and *Cucurbit aphid-borne yellows virus* (CABYV). (**A3**) A scheme describing the distribution of nucleotide sequences in the PeVYV genome that are very similar to sequences found in the homologous locations in the genomes of TVDV, CABYV and *Potato leaf roll virus* (PLRV). The putative recombination regions were identified by BLAST search analysis. The solid arrows indicate the positions of the putative recombination sites in ORF3, ORF4, ORF5 and the 3' NCRs of PYLCV and PeVYV.

BLAST-n and BLAST-p algorithms were used to compare the nucleotide and amino acid sequences (respectively) of PYLCV with other polerovirus sequences. The best hit for each ORF is presented in Table 2. The BLAST-p algorithms were applied to calculate the level of similarity between the amino acid sequence of each ORF of PYLCV and those of other poleroviruses, as summarized in Table 2. The highest levels of shared amino acid sequence identity between PYLCV and PeVYV ORFs range between 86% and 98%, while lower levels of amino acid sequence identity (55% to 93%) were observed when PYLCV was compared with TVDV (Table 2). These results were further confirmed by the alignments of the P0 (ORF0) (Figure S1 Table 2) and the MP (ORF4) deduced amino acid sequences of these viruses (Figure S2 Table 2). On the other hand, the deduced amino acid sequences of genome segments located at the N-termini of P5 (First 233 aa), sequences of PYLCV and PeVYV are very similar to CABYV, but not to TVDV or PLRV (Figure 2 and Figure S3 Table 2). Alignment analysis of the deduced amino acid sequences of PYLCV ORFs with those of PeVYV and TVDV demonstrated that the PYLCV ORFs (P3, P4, and P5) contain aa sequences that are very similar to those of PeVYV and TVDV (Figure 2 Table 2).

**Table 2:** Amino acid sequence identity (%) of *Pepper yellow leaf curl virus* (PYLCV) encoded by the six ORFs and homologous ORFs encoded by several established members of the genus *Polerovirus*.

ORF	Position in the PYLCV genome	TVDV	PeVYV	*PepYV	CABYV
ORF0	51-800	**77**	**85**	—	**32**
ORF1	175-2139	**78**	91	—	**36**
ORF2	1667-3431	93	96	—	**68**
ORF3	3631-4242	90	98	94	**65**
ORF4	3662-4123	**84**	**86**	90	**53**
ORF5	4243-5730	**55**	91	—	**87**

* Partially sequenced

The values in the table represent the levels of similarity (%) of the amino acid sequences for the major open reading frames encoded by *Pepper yellow leaf curl virus* (PYLCV) and those of other *Polerovirus* members: *Pepper vein yellows virus* (PeVYV) (accession no. AB594828), *Pepper yellows virus* (PepYV) (accession no. FN600344 partial sequence), *Tobacco vein-distorting virus* (TVDV) (accession no. EF529624) and *Cucurbit aphid-borne yellows virus* (CABYV) (accession no. AY529654). A grey background indicates less than 90% sequence similarity.

**Figure 3:**
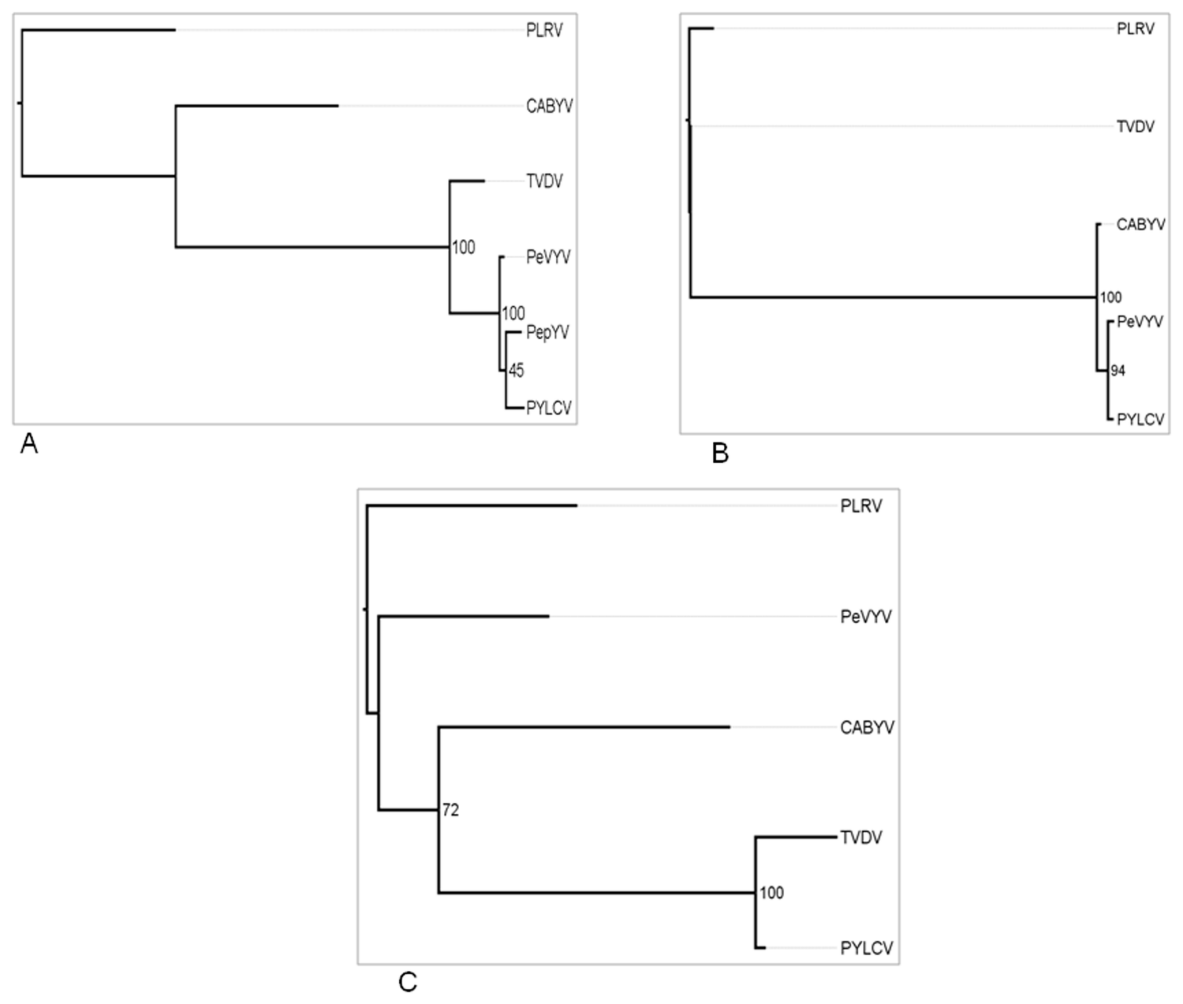
Phylogenetic analysis of selected poleroviruses based on the following proteins: **(A)** CP (ORF3), **(B)** the N-terminus of the RTD (ORF5, first 233aa) **(C)** the C-terminus of the RTD (ORF5, last 262aa) of *Potato leaf curl virus* [PLRV (Y07496)], *Tobacco vein-distorting virus* [TVDV (EF529624)], *Cucurbit aphid-borne yellows virus* [CABYV (X76931)], *Pepper vein yellows virus* [PeVYV (AB5948280)], *Pepper yellow leaf curl virus* [PYLCV (HM439608)], and *Pepper yellows virus* [(PepYV) FN600344]. Each protein-coding sequence was aligned using the MUSCLE program and phylogenetic trees were then constructed for each data set using the PhyML software with 100 bootstrap replicates and PLRV, the type member of the genus *Polerovirus*, as an out-group. The sequence of PepYV ORF 5 is not available and was not used for the construction of the phylogenetic trees described in B.

**Figure 4:**
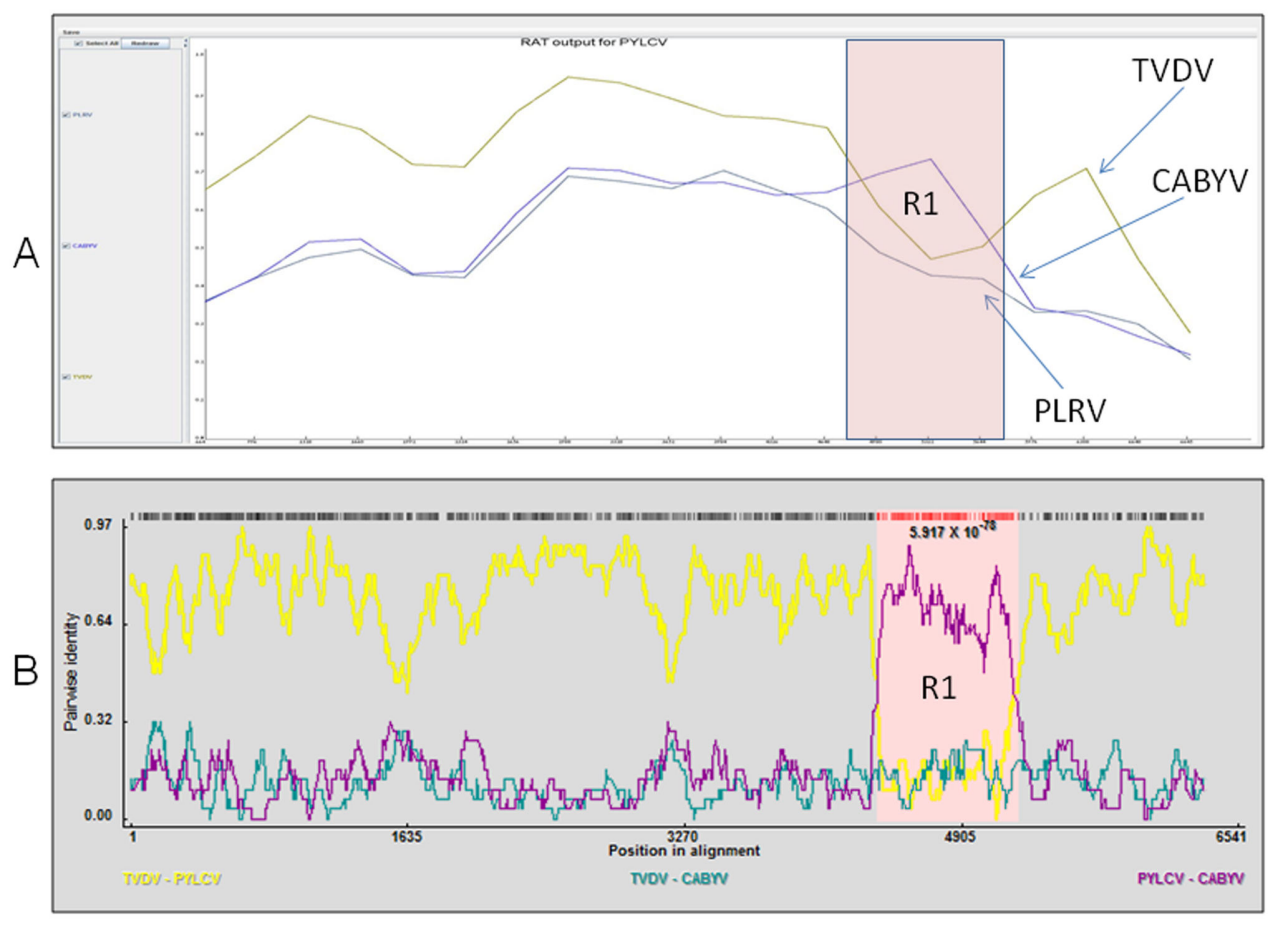
Presentation of the frequency of recombination event in the genomes of several poleroviruses, calculated using two different recombination detection methods. The position in the virus nucleotide sequence alignment is shown on the x-axis. The y-axis shows the percentage of pair-wise sequence identity. (**A**) Recombination Analysis Tool (RAT), R1, the peak in the pink zone represents a recombination event between *Pepper yellow leaf curl virus* (PYLCV) and *Cucurbit aphid-borne yellows virus* (CABYV) (accession numbers HM439608 and X76931, respectively). (**B**) RDP3 analysis R1, the peak in the pink zone represents a recombination event between *Pepper yellow leaf curl virus* (PYLCV) and *Cucurbit aphid-borne yellows virus* (CABYV) (accession numbers HM439608 and X76931, respectively) between positions 4227-4962 (*p*-value < e-77). The line colors represent the alignment between different pairs of poleroviruses: yellow - *Tobacco vein distorting virus* (TVDV) and PYLCV; violet - PYLCV and CABYV; green - TVDV and CABYV.

Phylogenetic analyses were performed on the deduced amino acid sequences of each of the individual PYLCV putative proteins: P1, P2, CP, and MP, as well as the N-terminus and C-terminus of PYLCV RTD protein (P5), using the Maximum Likelihood (ML) method. Comparison of PYLCV CP with each of the homologous proteins of several other poleroviruses, indicated a close phylogenetic relationship among PYLCV, PeVYV and PepYV. In contrast, a greater phylogenetic distance was found for the poleroviruses TVDV and CABYV (Figure 3A). These phylogenetic relationships were further confirmed for P1, P2 and MP (data not shown). However, the phylogenetic trees constructed based on the above mentioned putative proteins were completely different from the phylogenetic trees constructed on the basis of the putative recombined N and C termini of PYLCV RTD protein. The different phylogenetic topologies obtained from the different data sets (Figure 3) support the theory for hypothetical recombination event between CABYV and PYLCV ancestors. The evolution of PYLCV through recombination was further studied using the RDP3 package software [18]. The results of this analysis indicated the possibility of one major recombination event: between a PYLCV prototype and CABYV. This is further supported by the significant *p*-values (5.9e-78 and 1.8e-67) that were obtained in the RDP3 analysis (Figure 4). The hypothesized occurrence of the supposed recombination event is also supported by the highly significant *p*-values (*p* < 0.05) obtained in the analyses conducting using the GENECONV, Bootscan, MaxChi, Chimera and 3seq bioinformatic software programs.

## Discussion

PYLCV was found to be the causal agent of a new disease of pepper that has been spreading in Israel since 1998. PYLCV is transmitted by at least two aphid species, the green peach aphid *M* *. persicae* and the cucurbit aphid *A* *. gossypii* , in a persistent manner [3]. In the current study, the complete genomic sequence of PYLCV was obtained and used to predict the genome organization and its taxonomic affiliation within the genus *Polerovirus*, as well as its phylogenetic relationship with previously reported pepper-infecting poleroviruses and other viruses in the genus.

PYLCV has a typical polerovirus genome organization that includes six putative proteins encoded by the six ORFs (Figure 1). ORF0 encodes the protein P0, which has been reported to suppress RNA-silencing activity in poleroviruses [25,26,27]. ORF1 encodes the protein P1, which is known to exhibit serine protease activity in PLRV, the type member of the genus *Polerovirus* [28]. ORF2 overlaps ORF1 and is expressed through a -1 frame shift yielding a 65.58 kDa protein 2 (P2), which functions as an RNA-dependent RNA polymerase (RdRp) that is highly conserved in positive-stranded RNA viruses [29]. ORF3 encodes the 22.51 kDa major coat protein (CP), which overlaps ORF4 encoding the putative movement protein. ORF5 has a read-through domain (RTD), responsible for a 'read-through' protein with a MW of 77.94 kDa that produces the minor CP, which comprises the fused products of ORF3 (CP) and the adjacent ORF5 [30]. It has been suggested that the RTD protein, which is exposed on the surface of the virus particle, facilitates virus movement in infected plants [31,32] and is also involved in virus transmission [30,33].

The PYLCV genome includes three non-coding regions (NCR): the 5’ NCR, which is 50 nt in size; the 199-nt intergenic NCR located between ORF2 and ORF3 and a 298-nt NCR at the extreme 3' genome terminus (Figure 1), which is significantly shorter than the 402-nt-long 3' NCR reported for PeVYV [10]. The 5' end of the PYLCV genome starts with the typical polerovirus motif, ACAAAA [34]. The region between nucleotides 4,227 and 4,962 (ORF 5) has the highest level of similarity (78%) with the homologous region of the CABYV genome (Table 2 Figures 2A1, 2A2). A potential pseudo-knot structure with a frame-shifting slippery heptamer was identified at position 1652 of the PYLCV genome, slightly upstream to position 1667 where ORFs 1 and 2 overlap (Figure 1), starting with the conserved motif 5’-GGGAAAC-’ 3, previously reported for other poleroviruses [35,36]. The region located between nucleotides 3,631 and 4,226 of PYLCV (which includes most of the ORF3 and the entire ORF4) and the region between nucleotides 5,062 and 5,866 (ORF 5 and 3' NCR) have 85% nucleotide sequence identity with homologous regions of the TVDV genome [37] (Figure 2A1–A2).

Two additional regions unique to PYLCV (PY1 and PY2) were identified between nucleotides 4,962 and 5,061 (ORF 5) and between nucleotides 5,866 and 6,028 (3' NCR). These sequences do not share sequence identity with any virus sequence in the GenBank. Both regions share low levels of identity with the partial homologous sequence of PeVYV (Figure 2A2 Table 2). Similarly, sequence analysis of the pepper-infecting PeVYV revealed three unique regions (Pe1-Pe3) not found in other members of the genus *Polerovirus*. Pe1 is located between nucleotides 5,002 and 5,362 (361 nt at the core region of ORF5), similar to the PY1 position in PYLCV ORF 5. Pe2 is located between nucleotides 5,561 and 5,945 (384 nt at the C-terminus of the RTD protein (ORF 5) and Pe3 is located between nucleotides 6,038 and 6,244 (206 nt at the 3’ NCR), similar to the position of PY2 in the PYLCV genome (Figure 2B). These two unique regions (PY2 and Pe3) were found to have specific stem-loop structures required for the replication and accumulation of RNA of luteoviruses [38]. The PeVYV ORF 5 contains a PLRV-like region (76% identity) between nucleotides 5,362 and 5,561. These unique regions found in both PYLCV and PeVYV may originate by a recombination event from an unknown common ancestral polerovirus. It can be assumed that these unique sequences found in ORF5 may act as determinants of host range and/or affect virus interaction with the vector and may explain the differences in host range between PeVYV and PYLCV [3,10].

A BLAST search analysis of the deduced amino acid sequences encoded by individual PYLCV ORFs (ORF0-ORF5) revealed high levels of sequence identity with the homologous PeVYV ORFs [85%, 91%, 96%, 98%, 86% and 91%, respectively] and with TVDV ORF2-ORF4 [93%, 90% and 84%, respectively]. Relatively low levels of shared sequence identity were calculated when these sequences were compared to the ORF0, ORF1 and ORF5 sequences of TVDV (77%, 78% and 55%, respectively). The lowest levels of shared sequence identity were calculated for PYLCV and CABYV ORFs (ORF0-ORF5), with the exception of ORF5, for which there was a relatively high level of shared sequence identity (87%) (Table 2).

Based on the complete genome sequences and the genome organization described above, PYLCV (accession number HM439608) is a putative member of the genus *Polerovirus* in the family *Luteoviridae* [3,10]. The two reported pepper-infecting poleroviruses, PYLCV and PeVYV, have very similar nucleotide and amino acid sequences (Figures 2 and 3 Table 2). Both viruses are capable of infecting pepper plants and induce similar symptoms. However, according to the Ninth Report of the International Committee on Taxonomy of Viruses [39], a threshold 10% difference in amino acid identity in any of the viral proteins is a criterion for the classification of a virus as a distinct species within the family *Luteoviridae*. ORF0 and ORF4 of PYLCV share 85% and 86% amino acid sequence identity with PeVYV (14-15% difference), supporting the identification of PYLCV as a distinct species in the genus *Polerovirus*.

The presence of viral genomic domains with very similar sequences among viruses in the family *Luteoviridae* is well documented [34,36]. This unique characteristic theoretically allows recombination event to occur between different members of the family. The specific sites in the genome that are involved in recombination are referred to as recombination breakpoints [40]. In several poleroviruses including CABYV, the recombination breakpoint is located at ORF3, which encodes the viral CP [40]. This may explain the CABYV-like sequences found in the genomes of PYLCV and PeVYV (Figure 2). As shown in Figure 2, ORFs 3, 4 and 5 on the 3' halves of the PYLCV and PeVYV genomes are composed of mosaics of sequences that are very similar to those of TVDV and CABYV, suggesting that recombination event have occurred during mixed infections of common host plants.

CABYV-like sequences that have been identified in the PYLCV genome and include the distal part of ORF3 and 265 amino acids of ORF5 coding for the read-through (RT) protein [30,41] may explain the ability of PYLCV to infect squash (*Cucurbita pepo*). This CABYV-derived sequence that was identified in the PYLCV minor CP (Figure 2) is probably responsible for the serological cross-reaction between PYLCV and CABYV in both ELISA and western blot assays [3] using polyclonal CABYV antiserum [42]. The difference in the host ranges of PYLCV and PeVYV is demonstrated by the ability of the first to infect *Datura* *stramonium* and *Petunia hybrida* while PeVYV is not infecting *D* *. stramonium* [9] *P. hybrida* was not tested for infection of PeVYV but reported as a host of TVDV [37].

Sequence analysis of the complete genomes of PYLCV and TVDV revealed the presence of TVDV-like regions in ORFs 3, 4 and 5 of PYLCV, which may reflect the significant difference between the host ranges of these viruses. Phylogenetic analysis has allowed the study of the likelihood of horizontal gene transfer events that may have occurred via recombination events during the evolution of poleroviruses. Based on a phylogenetic analysis using the CP, of the pepper-infecting poleroviruses PYLCV, PeVYV and PepYV, it is suggested that these viruses evolved from TVDV and, as shown in Figure 3A, they are all clustered together on a separate branch of the phylogenetic tree. Similar phylogenetic relationships were reported by Murakami et al. (2011) [10].

In a different phylogenetic analysis using the 5' and 3' ends of ORF 5, it is demonstrated that the separation of PYLCV and PeVYV from their putative parental TVDV probably occurred through an earlier recombination event (Figure 3B Figure S4). This recombination event may have opened a large number of options for the creation of a virus with novel characteristics, such as an expanded host range or modified vector specificity [34,43,44]. Indeed the host range reported for PYLCV is significantly broader than that of TVDV [3].

Different topologies of the phylogenetic trees were obtained when separate analyses of the 5' end and 3' end of ORF5 were performed (Figure 3B, C Figure S4) versus a tree that was obtained from the analysis of ORF 3 (Figure 3). This difference is an indicator for one putative recombination event, which presumably occurred before the evolutional splitting of PYLCV and PeVYV by an introduction of a CABYV-like sequence into their genomes. Alternatively this scenario can be explained by the introduction of PLRV-like sequence into the PeVYV genome (Figure 4). It can be assumed that beside the suggested recombination scenarios the resulting sequence differences were formed by both positive and purifying selection constraints acting at the protein and genome level. Additional information from genome sequencing of poleroviruses may shed light on the evolutionary process underling these sequence differences.

Evolutionary studies of viruses within the *Luteoviridae* indicate that the *Luteovirus* and *Polerovirus* genera originated around 1500 years ago. Luteovirus species appeared within the last 500 years, similar to other families of plant RNA viruses. It has been hypothesized that the intensification of agriculture, which resulted in increased populations of cultivated plant varieties, as well as the establishment of global communication networks have affected the extent and structure of genetic variation in many plant RNA viruses [40]. This suggested evolutionary process is supported by the reported outbreaks of pepper diseases caused by poleroviruses in Israel [3], Japan [10], Turkey, Tunisia [4] and recently in India, Indonesia, Mali, Philippines, Taiwan, and Thailand [5]. The emergence of pepper-infecting poleroviruses coincides with the dramatic increase in the size of pepper markets in Israel and other Mediterranean countries. Future studies of the entire genomes of these newly reported pepper poleroviruses may shed light on the evolution of this group of viruses, as well as the function(s) of specific sequence elements that control their host range.

## Supporting Information

Figure S1 Alignment of the deduced amino acid sequences corresponding to the ORF0s of Pepper *yellow leaf curl virus* [PYLCV (HM439608)] and the two poleroviruses *Pepper vein yellows virus* [PeVYV (AB5948280)] and *Tobacco vein-distorting virus* [TVDV (EF529624)].
(TIF)

Figure S2 Alignment of the deduced amino acid sequences of the ORF4s of the poleroviruses *Pepper yellow leaf curl virus* [PYLCV (HM439608)], *Pepper vein yellows virus* [PeVYV (AB5948280)], *Pepper yellows virus* [PepYV (FN600344)], *Tobacco vein-distorting virus* [TVDV (EF529624)], *Potato leaf curl virus* [PLRV (Y07496)] and *Cucurbit aphid-borne yellows virus* [CABYV (X76931)].
(TIF)

Figure S3 Alignment of the deduced amino acid sequences of the N-termini of the ORF5s (CABYV-like region) of the poleroviruses *Pepper yellow leaf curl virus* [PYLCV (HM439608)], *Pepper vein yellows virus* [PeVYV (AB5948280)], *Cucurbit aphid-borne yellows virus* [CABYV (X76931)], *Potato leaf curl virus* [PLRV (Y07496)] and *Tobacco vein-distorting virus* [TVDV (EF529624)].
(TIF)

Figure S4 Phylogenetic analysis of selected poleroviruses based on the deduced amino acid sequences of the ORF5 of *Potato leaf curl virus* [PLRV (Y07496)], *Tobacco vein-distorting virus* [TVDV (EF529624)], *Cucurbit aphid-borne yellows virus* [CABYV (X76931)], *Pepper vein yellows virus* [PeVYV (AB5948280)], *Pepper yellow leaf curl virus* [PYLCV (HM439608)]. Each protein-coding sequence was aligned using the MUSCLE program and phylogenetic trees were then constructed for each data set using the PhyML software with 100 bootstrap replicates and PLRV, the type member of the genus *Polerovirus*.
(TIF)
